# Reactive MD Screening of Antioxidants for Substituent-Dependent Phenoxyl Radical Stability

**DOI:** 10.1021/acsomega.6c00592

**Published:** 2026-03-02

**Authors:** Shihab Ahmed, Stefan J. Eder, Mohamed Musthafa Iqbal, Nicole Dörr, Ashlie Martini

**Affiliations:** † Department of Mechanical Engineering, University of California, Merced, 5200 N. Lake Road, Merced, California 95343, United States; ‡ 333143 AC2T research GmbH, Viktor-Kaplan-Straße 2/C, Wiener Neustadt 2700, Austria; § 27259 Institute of Engineering Design and Product Development, TU Wien, Lehárgasse 6 - Objekt 7, Vienna 1060, Austria

## Abstract

Oxidation limits the performance and lifetime of lubricants, and phenolic antioxidants are commonly used to slow this process by scavenging hydrocarbon peroxyl radicals. The performance of phenolic antioxidants is largely determined by the stability of the antioxidant radical that remains after hydrogen donation. To explore the relationship between antioxidant chemical structure and radical stability, we used REACTER-based reactive molecular dynamics simulations to model the reverse hydrogen transfer reaction from polyalphaolefin hydroperoxides to phenoxyl radicals. Simulations were run for 718 distinct single-ring phenoxyl radicals with varied substituent types and positions in a polyalphaolefin hydroperoxide environment. Reaction rates were obtained from the time evolution of hydrogen transfer events, where lower reaction rates correspond to higher radical stability and better antioxidant performance. Analysis of diffusivity, hydrogen bonding, and steric hindrance showed that strong hydrogen bonding and high steric hindrance around the phenoxyl oxygen atom decreased the reaction rate, while faster diffusion increased it. A multivariate linear model confirmed that hydrogen bonding was the dominant contributor to radical stability in the low reaction rate region. These results highlight the molecular features that influence antioxidant behavior and demonstrate that reactive simulations offer an efficient route for screening and designing antioxidant molecules.

## Graphical abstract

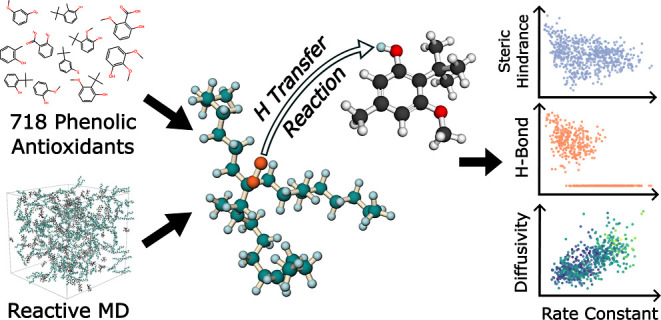

## 1 Introduction

As mechanical systems become increasingly sophisticated, the demand for higher-performance lubricants continues to grow. One of the primary obstacles to improving lubricant performance is oxidation, a process initiated and propagated by free radical chain reactions.^1^ This process is significantly accelerated under harsh operating conditions, including elevated temperatures, high pressures, and direct metal-to-metal contacts.^2^ To slow oxidation and extend the useful lubricant life, lubricant formulations typically incorporate antioxidant additives. These additives act through various mechanisms, particularly as radical scavengers (e.g., sterically hindered phenols, aromatic amines), peroxide decomposers (e.g., sulfur- and phosphorus-based compounds), and metal deactivators (e.g., benzotriazoles).^3^

The antioxidant performance of lubricants is evaluated through thermal-oxidative stability tests that determine their resistance to degradation under heat and oxygen-rich conditions.^2^ Widely used experimental techniques include differential scanning calorimetry, thermogravimetric analysis, oxidation onset temperature measurements, and rotary bomb oxidation tests, which quantify changes such as shifts in oxidation onset or reduction in mass at elevated temperatures.^4–8^ For example, a study demonstrated that combining differential scanning calorimetry with oxygen pressure tests provides complementary insights into oxidation resistance and the effectiveness of antioxidant additives.^4^ Other work has explored nonisothermal thermogravimetric analysis to derive kinetic parameters and oxidation induction times, enabling a faster and quantitative evaluation of lubricant oxidative stability.^5,9^ While these approaches are effective for characterizing bulk oxidation behavior, they provide limited insight into the molecular-level mechanisms governing antioxidant activity, which are crucial for understanding structure-performance relationships and for guiding the rational design of improved lubricant additives.

To overcome this limitation, molecular dynamics (MD) simulations can be a powerful tool for studying antioxidants in lubricants at the atomic scale, although only a few studies have applied this approach to date.^10–14^ Nonreactive MD studies, which model atomic motion without allowing bond breaking or formation, have examined the diffusion and distribution of antioxidants within base oils. Such simulations have provided insights into antioxidant mobility and how it influences their ability to intercept radicals and delay oxidation. Prior work has shown that antioxidants with larger molecular weights and longer alkyl chains exhibit lower mobility yet provide greater resistance to oxygen permeation, resulting in enhanced oxidative stability of the base oil.^13^ Similar studies on ester-based lubricants with ferulic acid derivatives show that antioxidant performance depends on solubility, dispersion, and oxygen permeability.^12^ Additionally, nonreactive MD methods have also been applied in the design and evaluation of novel lubricant additives, where binding interactions with surfaces such as steel or diamond-like carbon coatings were characterized to predict antioxidant performance.^15^ However, nonreactive MD cannot capture chemical reactions, which are essential for understanding how antioxidants scavenge radicals and inhibit oxidation.

Reactive MD, which allows bond breaking and formation to be simulated directly based on reactive force fields, has been widely used to study chemical processes in tribochemistry.^16^ Among these, ReaxFF,^17^ a widely used reactive MD force field, has been extensively applied to model oxidation and degradation reactions in organic^18,19^ and interfacial systems^20^ as well as to investigate lubricant oxidation behavior.^21–23^ For example, one study examined the oxidation behavior of ester-based lubricants under high-temperature, oxygen-rich conditions and found that tri-isodecyl trimellitate (TDTM) exhibited higher oxidation resistance than di-isooctyl adipate (DOA), with simulations showing fewer bond cleavages and degradation products in TDTM compared to DOA.^21^ Given its ability to capture oxidation chemistry, ReaxFF appears to have strong potential for studying lubricant antioxidants, though its application in this context has been limited. It has already been used to explore oxidation processes in vegetable oils and other food-related systems. For instance, one study revealed that antioxidants protect oils by releasing hydrogen atoms to scavenge free radicals, thereby inhibiting β-scission reactions and slowing oil degradation.^24^ It further demonstrated that butylated hydroxy­toluene and *tert*-butyl hydroquinone are the most effective antioxidants, whereas butylated hydroxyanisole and propyl gallate show lower effectiveness, with performance influenced by the concentration and oxygen content. However, even though ReaxFF has the potential to be used for lubricant antioxidants, it remains limited by its system-specific parametrization, which requires extensive tuning for each new system.^25^ Moreover, simulations with ReaxFF are significantly more computationally expensive than those with nonreactive force fields,^26^ making it challenging to perform longer simulations needed to obtain reliable reaction statistics for antioxidant mechanisms.

As an alternative to fully reactive force fields, REACTER extends classical nonreactive MD by enabling the simulation of predefined chemical reactions through dynamic updates of molecular topology during runtime.^27^ This approach has been successfully employed to investigate polymerization and cross-linking processes, demonstrating its ability to capture complex reaction networks within large-scale classical MD simulations.^28–31^ Because REACTER is built on classical MD, it offers substantially higher computational efficiency than ReaxFF and does not require system-specific parameter tuning as it relies on predefined reaction rules rather than fitted reactive parameters. This efficiency enables the study of larger systems and longer time scales, which are essential for collecting statistically meaningful behavior and exploring diverse antioxidant structures. However, REACTER has not yet been used to study lubricant antioxidants.

In this study, we used the REACTER protocol within MD simulations to investigate the relationship between chemical structure and antioxidant function, focusing on phenolic antioxidants. The simulations quantified the rate of hydrogen transfer from hydroperoxides of polyalphaolefin (PAO) base oil to phenoxyl radicals, where slower reaction rates correspond to more stable radicals and better antioxidant performance. We created 718 single-ring phenoxyl radicals with different ring substituents, allowing us to test how both the substituent type and position influence antioxidant-derived radical stability. We then evaluated the influence of diffusion, hydrogen bonding (H-bonding), and steric hindrance on the radical stability. Finally, a multivariate regression was used to determine the key contributing factors. Using this framework, we evaluated stability trends across a broad chemical range of antioxidant-derived radicals.

## 2 Methods

### 2.1 Reaction Mechanism

Phenolic antioxidants function as primary antioxidants by donating a hydrogen radical from their hydroxyl group to scavenge reactive peroxyl radicals (ROO^•^) on a hydrocarbon chain, thereby interrupting the propagation stage of lubricant oxidation. After this process, the antioxidant itself is converted into a phenoxyl radical, while the peroxyl radical is reduced to a hydroperoxide (ROOH). The generated antioxidant radical can revert to the original molecule, so the stability of the radical is a crucial property for effective antioxidation. Since radical stability is a key indicator of phenolic antioxidant performance,^32^ this is the focus of our simulations.

In this study, the hydroperoxides were derived from the PAO base oil, a synthetic hydrocarbon commonly used in high-performance lubricants due to its high viscosity index and excellent low-temperature performance.^33^ A tertiary carbon site was selected to attach the −OOH group to form hydroperoxide since tertiary carbons form more stable radicals.^2^ The use of PAO-derived hydroperoxides enables the simulated oxidation and antioxidant reactions to closely replicate those observed in lubricant degradation processes, ensuring the relevance and applicability of the modeling to real-world formulations. The PAOs were modeled as trimers of 1-decene, corresponding to the PAO 4 grade used in lubricant formulations.^34,35^ Here, we modeled the reverse hydrogen transfer reaction in which PAO hydroperoxides react with antioxidant phenoxyl radicals, producing antioxidant molecules and PAO peroxyl radicals as products. The structures of the reactants and products are shown in Figure 1.

**Figure 1:**
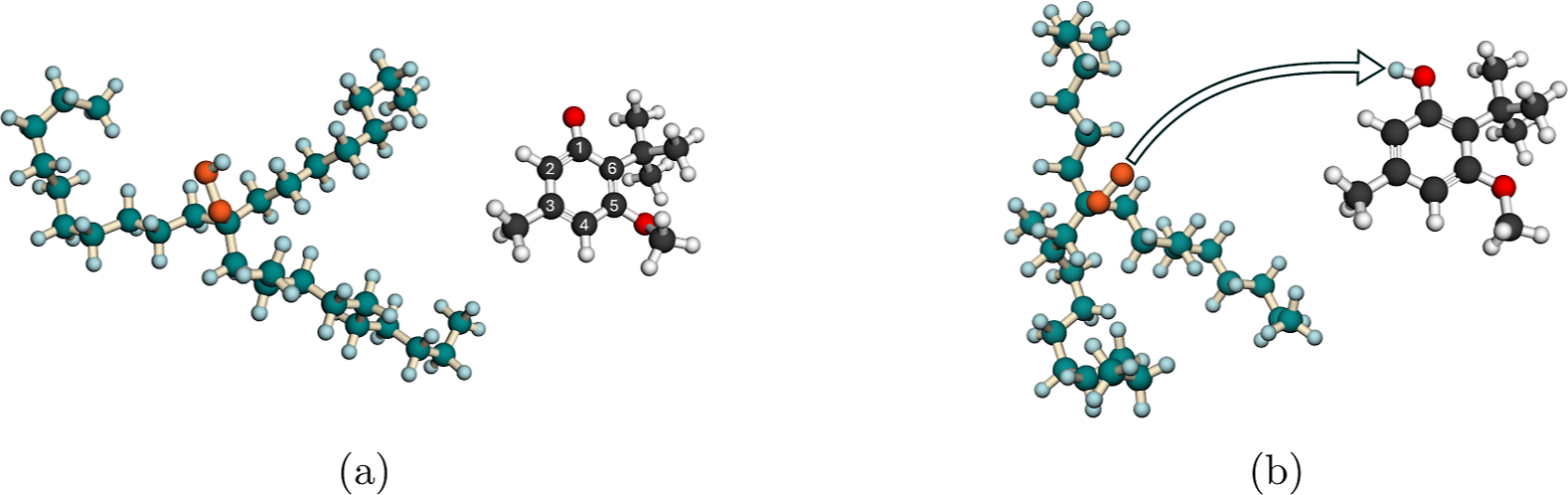
Molecular structures of (a) the reactants, PAO hydroperoxide and 2-*tert*-butyl-3-methoxy-4-methylphenoxyl radical, and (b) the products, PAO peroxyl radical and 2-*tert*-butyl-3-methoxy-4-methylphenol. The arrow on the product structures indicates the origin and final position of the transferred hydrogen. In PAO derivatives, carbon atoms are represented in dark cyan, oxygen in orange, and hydrogen in light blue. In antioxidants, carbon atoms are represented in dark gray, oxygen in red, and hydrogen in white.

### 2.2 Generation of Phenolic Antioxidant Candidates

Candidate antioxidant molecules were generated combinatorially using the phenoxyl radical as the base scaffold, with the oxygen atom fixed at position 1 of the aromatic ring. Substituent positions were varied to systematically explore the structural diversity of phenolic derivatives. The substituent set included hydrogen (H), *tert*-butyl (*t*Bu), methoxy (OMe), methyl (Me), and carboxyl (COOH) groups. This combinatorial scheme yielded a total of 1625 unique phenoxyl radical substitution patterns. After combinatorial generation, structure-based filtering was applied to remove chemically unrealistic configurations. The following criteria were used:

1. *t*Bu and COOH substituents were limited to a maximum of two each.
2. Molecules in which two *t*Bu groups were placed at adjacent positions or a *t*Bu group was adjacent to a COOH group were excluded.
3. Ortho positions (positions 2 and 6) carrying both a *t*Bu and a COOH group simultaneously were not considered.
4. The total number of substituents on the ring was restricted to four or fewer.

These constraints reduced the candidate pool to 718 radicals, which were subsequently used for molecular dynamics simulations.

The distribution of the molecular masses of all 718 radicals is shown in Figure 2a and is slightly left-skewed, because heavier substituents were restricted more strictly than lighter ones. Figure 2b shows the distribution of substituent types across positions 2 to 6 of the ring among the generated radicals; substituent positions are defined in the inset of Figure 2b. These radicals were represented as SMILES strings,^36^ which were then used as inputs for MD simulations.

**Figure 2:**
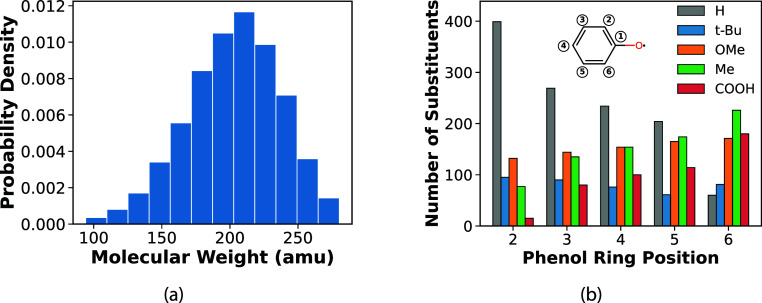
(a) Distribution of molecular weights for the antioxidant radicals. (b) Number of substituents at each position on the phenoxyl ring, grouped by substituent type. The inset shows the numbering scheme used for the phenol ring positions.

### 2.3 Molecular Dynamics Simulations

We used the LUNAR^37^ program to set up the REACTER simulations. The preparation involved several steps, including atom typing, assigning force field parameters, generating the reaction map file, and creating the bulk system by randomly placing the reactants inside the simulation cell. To streamline this process, we implemented an automated script that takes the SMILES strings of the antioxidant radical candidates and the PAO hydroperoxide as input and runs the LUNAR scripts for each system to generate the initial bulk system for the MD simulations.

Each simulation cell was constructed by randomly dispersing 100 antioxidant radicals and 125 PAO hydroperoxides under periodic boundary conditions, as shown in Figure 3. Although the concentration of antioxidants is higher than what is found in real lubricant formulations, this was done to ensure that a statistically significant number of hydrogen transfer events could be observed within the limited simulation time. Three independent realizations were prepared, each with distinct initial atom positions and velocities to improve statistical sampling. All systems were first energy-minimized and then equilibrated in the isothermal–isobaric (NPT) ensemble at 300 K and 1 atm. During this stage, the density was continuously monitored, and equilibration was terminated once the density reached a stable value, indicating convergence. The systems were subsequently equilibrated in the canonical (NVT) ensemble at 300 K for 500 ps, which was sufficient to stabilize energy fluctuations, as confirmed by monitoring the convergence of potential energy profiles. Temperature and pressure control were maintained using a Berendsen thermostat and barostat with a damping parameter of 100 fs, where the time step was set to 1 fs. During these equilibration stages, all predefined reactions were disabled to ensure that only structural and thermodynamic stabilization occurred prior to initiating reactive simulations. The reaction simulations were then run for 2 ns with a time step of 1 fs.

**Figure 3:**
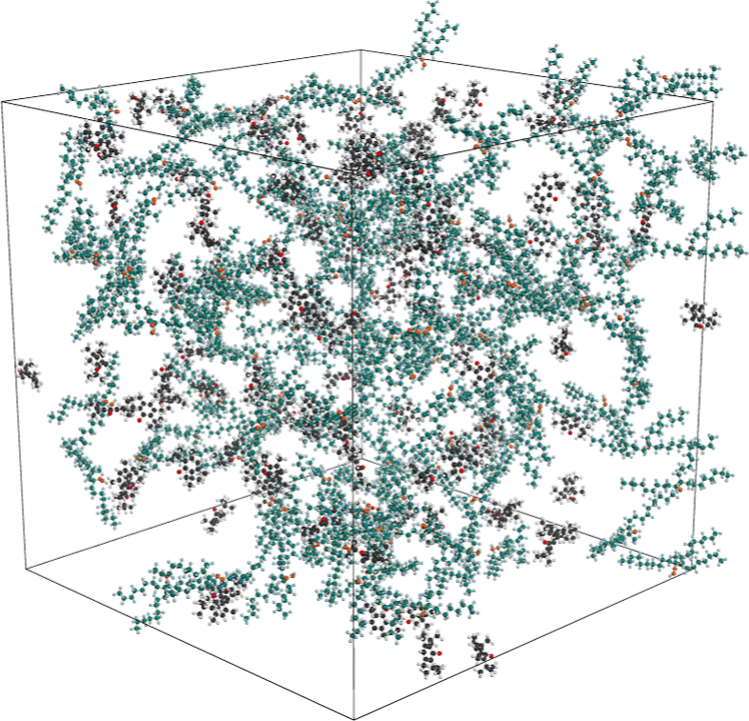
Initial configuration of the simulation box containing 100 phenoxyl radicals from antioxidants and 125 PAO hydroperoxides. Atom colors follow the same scheme as in Figure 1.

The reaction criterion was distance-based, with a reaction triggered when the hydrogen atom in the PAO hydroperoxide approached within 0.9 Å of the phenoxyl oxygen atom. This cutoff is close to the typical O–H distances reported for transition states in similar hydrogen atom transfer reactions (≈1.1–1.4 Å from CBS-QB3 calculations^38^), allowing the simulation to capture a significant number of hydrogen-transfer events.

We quantified the stability of an antioxidant radical by the rate at which hydrogen-transfer reactions occur; a lower rate constant indicates a more stable antioxidant radical. To obtain this rate constant, we fit an exponential function to the average reaction-count trajectory from three independent realizations,

$$N\left(t\right)=N_{\infty }\left(1-e^{-kt}\right)$$

where *N*(*t*) is the cumulative number of reactions at time *t*, *N*_∞_ is the asymptotic plateau, and *k* is the effective rate constant. Because the saturation of *N*(*t*) arises solely from the finite number of available reactant molecules in the simulation, this functional form is not intended to represent the underlying reaction mechanism; rather, it provides a consistent numerical way to extract the effective rate constant used as our stability metric.

## 3 Results and Discussion

Figure 4a shows the evolution of mean reaction counts over time for three representative antioxidant systems, where the antioxidant radicals are displayed in the same color shade. The fitted curves yield system-specific rate constants, which are taken as the performance metric. A histogram of the extracted rate constants for the full data set of antioxidants is shown in Figure 4b. The results identify a subset of antioxidants with lower rate constants, indicating higher radical stability. We next examined how the performance metric relates to some physical properties, specifically, diffusion, H-bonding, and steric hindrance around the reaction site in the antioxidants.

**Figure 4:**
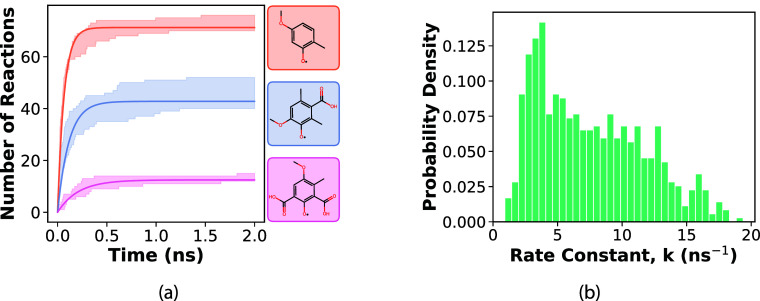
(a) Evolution of the mean number of hydrogen transfer reactions over time for three representative antioxidant systems. Each curve is color-coded to match its molecular structure shown on the right. The fitted curves yield the rate constant, which serves as the performance metric for assessing antioxidant stability. (b) Histogram of the extracted rate constants for all antioxidant systems.

### 3.1 Diffusivity

Since the hydrogen transfer reaction is fast, the reaction rate is controlled by diffusive encounters with radical species.^39,40^ To calculate the diffusivity, we analyzed the mean squared displacement (MSD) of the antioxidant radicals during the equilibration stage after the system energy had stabilized. A straight line was fitted to the linear portion of the MSD–time curve to calculate the diffusion coefficient using the Einstein relation.^41^ This calculation was done for each of the three simulations with each radical independently, and the final diffusion coefficient was determined as the average of these three values.

As illustrated in Figure 5, there is a positive trend between the rate constant and the diffusion coefficient, indicating that faster-diffusing radicals are statistically more likely to undergo a reaction, consistent with previous observations.^39,40^ Since we modeled the reverse hydrogen transfer reaction, a higher rate constant indicates lower antioxidant radical stability and is therefore detrimental. The diffusion coefficient is generally related to molecular size, with larger molecules exhibiting lower diffusion.^42^ This trend is also observed in Figure 5, where the symbol color represents the molecular weight, showing that heavier radicals generally diffuse more slowly and yield smaller rate constants. However, the observed scatter suggests that diffusion alone cannot fully account for the variations in stability, implying that other properties must also play a significant role.

**Figure 5:**
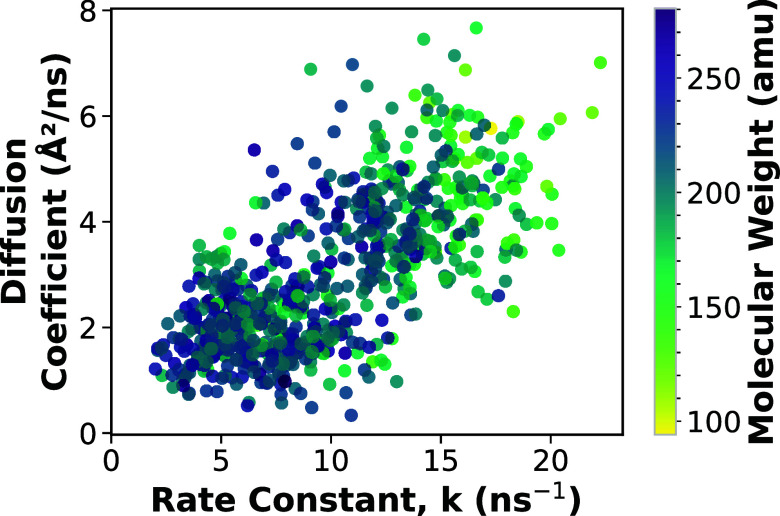
Diffusion coefficient as a function of rate constant. Each symbol represents the average diffusion coefficient for a given antioxidant system. The color scale denotes the molecular weight of the antioxidant radical. The results show that higher-molecular-weight radicals have slower diffusion and lower reaction rate.

### 3.2 Hydrogen Bonds

Hydrogen-bond (H-bond) formation is known to stabilize antioxidant radicals and therefore be beneficial for antioxidant performance.^43,44^ In the simulations, H-bonds were identified using the MDAnalysis^45^ python package based on two geometric criteria: (1) a donor–acceptor distance shorter than 3 Å and (2) a donor–hydrogen–acceptor angle greater than 150°. H-bonding was possible within a single radical, between different antioxidant radicals, and between antioxidant radicals and PAO hydroperoxides. For each system, the H-bond probability for a given radical was defined as the fraction of trajectory frames in which the phenoxyl oxygen site was protected by at least one H-bond. Trajectories were saved every 4 ps over a total simulation time of 2 ns, resulting in 500 uniformly spaced frames per simulation. These probabilities were averaged over all radicals in the system and over three independent simulations. To relate H-bonding to the overall reactivity, we compared the H-bond probabilities with the corresponding rate constants for each antioxidant system. The results are shown in Figure 6. Overall, the plot shows a decreasing trend: systems with higher H-bond probabilities have lower rate constants, whereas systems with lower H-bond probabilities have higher rate constants. This indicates that H-bonding generally contributes to radical stabilization by lowering the likelihood of reaction events, consistent with literature.^43,44,46^ There are also two clusters of data in Figure 6, indicating two groups of radicals with distinctly different H-bond probabilities associated with their substituent patterns.

**Figure 6:**
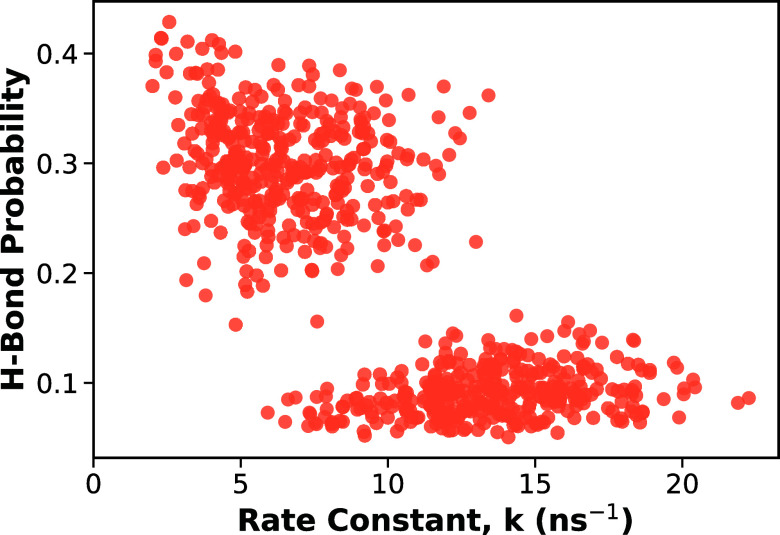
H-bond probabilities as a function of rate constant. Each symbol represents the H-bond probabilities for a given antioxidant system. The results show that antioxidant radicals with more H-bonds exhibit lower rate constants and are therefore more stable.

To evaluate which substituents contribute most to H-bonding, antioxidant radicals were grouped by the presence of specific position–substituent combinations, and the mean H-bond probability was computed for each group, as shown in Figure 7. Here, multiple groups can contain the same radical; for example, a radical bearing an ortho–COOH and a meta–Me substituent is included in both the ortho–COOH and meta–Me groups. Radicals containing COOH substituents exhibit the highest H-bond probabilities across all positions, with ortho–COOH showing the strongest association, followed by para–and meta–COOH. This trend is consistent with the fact that OH in the carboxyl group can act as a donor, while the phenoxyl oxygen atom can serve as an H-bond acceptor. In contrast, other substituents (Me, OMe, and *t*Bu) display substantially lower H-bond probabilities. Although these substituents do not directly participate in H-bonding, they appear to enhance the ability of the carboxyl group to do so, with methyl having the highest influence and *tert*-butyl having the lowest. This clear separation in H-bond probabilities between COOH-containing and non-COOH antioxidants gives rise to the two distinct clusters observed in Figure 6.

**Figure 7:**
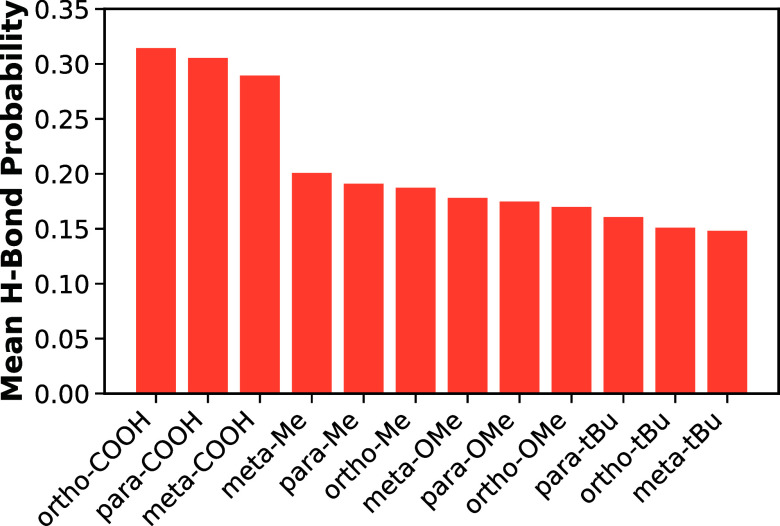
H-bonding behavior across position–substituent combinations. Each bar represents the mean H-bond probability for all antioxidant radicals containing a given position–substituent combination. A given radical may contribute to multiple bars if it contains more than one substituent.

### 3.3 Steric Hindrance

Steric effects provide additional stabilization for antioxidant radicals because bulky substituents can physically shield the reaction site and reduce its likelihood of undergoing further reactions.^44,47^ To quantify steric hindrance, we calculated the buried volume surrounding the phenoxyl oxygen atom, which serves as the reaction site.^48^ Buried volume measures the fraction of a defined spherical region around the phenoxyl oxygen atom that is occupied by the van der Waals volumes of nearby atoms, providing a direct assessment of how effectively the site is sterically protected. These calculations were performed using the MORFEUS package.^49^ For each antioxidant radical, the buried volume was computed from the trajectories generated during the reaction simulations. A sphere of radius 0.35 nm centered at the phenoxyl oxygen atom was used for this calculation. This radius was selected to include atoms bonded at the ortho positions while excluding more distant atoms that do not meaningfully contribute to steric effects at the reaction site. Finally, we obtained the time-averaged buried volume for each antioxidant simulation and then further averaged these values over the three independent simulations for each antioxidant system. The time-averaged buried volume captures two sources of steric hindrance: the intraradical component and the surrounding component. The intraradical steric hindrance comes from bulky functional-group atoms that stay close to the radical and move very little because they are bonded to the ring; therefore, this contribution remains nearly constant throughout the simulation. The surrounding steric hindrance, on the other hand, arises from nearby molecules and is much more dynamic, changing over time as those atoms move more freely.

The calculated buried volume values, expressed as percentages of the defined sphere, are plotted against the rate constant in Figure 8. A general decreasing trend is observed in which radicals with greater steric hindrance tend to exhibit lower rate constants, whereas those with less steric hindrance show higher rate constants. This observation is consistent with previous reports where sterically hindered phenoxyl radicals exhibit enhanced stability due to restricted access to the reactive site.^44,50^ These findings indicate that steric hindrance contributes meaningfully to the radical stability by limiting access to the reactive radical site.

**Figure 8:**
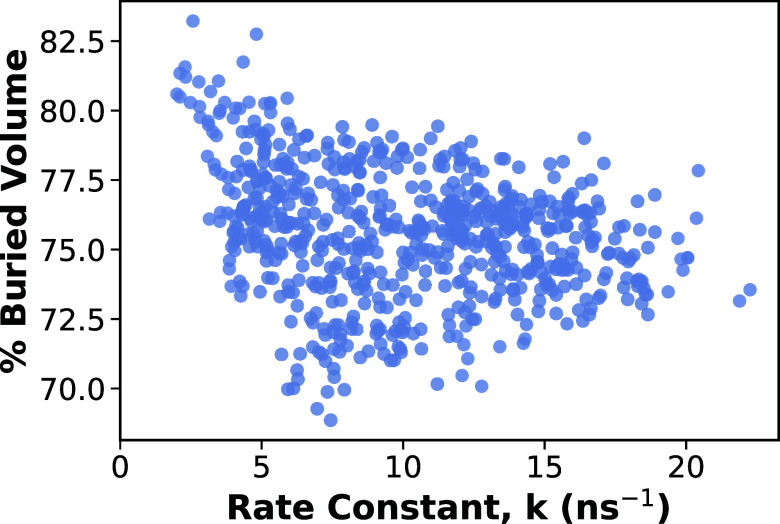
Buried volume as a function of the reaction rate constant. Buried volume is reported as a percentage of the defined sphere, and higher buried volume percentage corresponds to greater steric hindrance. Each symbol represents one of the 718 antioxidant radicals. The results show that radicals with higher buried volumes, indicating greater steric hindrance around the reaction site, exhibit lower rate constants and are therefore more stable.

To examine the steric hindrance of the radical site, we focused on the ortho positions (C2 and C6) adjacent to the phenolic oxygen, since substituents at these sites are positioned to directly block access to the phenoxyl oxygen atom. Figure 9 presents steric maps of all ortho–ortho combinations, visualized as top views of the spherical region centered on the phenoxyl oxygen, with the viewing plane perpendicular to the C–O bond (where O is the phenoxyl oxygen and C is the aromatic carbon bonded to it). To focus only on the antioxidant structure, atoms from the surrounding environment are removed from the maps. The maps are color-coded according to the vertical displacement relative to the central plane of the projection, with yellow regions corresponding to atoms protruding above the plane and purple regions indicating atoms lying below it. The percentage of the buried volume is shown in each panel. Radicals featuring small ortho substituents, such as H/H, display the lowest steric hindrance (68.6%), leaving the radical site highly exposed. In contrast, bulky *tert*-butyl groups provide extensive shielding, with *t*Bu and *t*Bu reaching the highest value (80.2%). Mixed or moderately sized substituents show intermediate effects; for example, H/Me achieves 70.6%, *t*Bu/Me reaches 73.5%, and OMe/COOH gives 78.5%. Together, these data confirm that steric hindrance increases systematically with the size and branching of ortho substituents.

**Figure 9:**
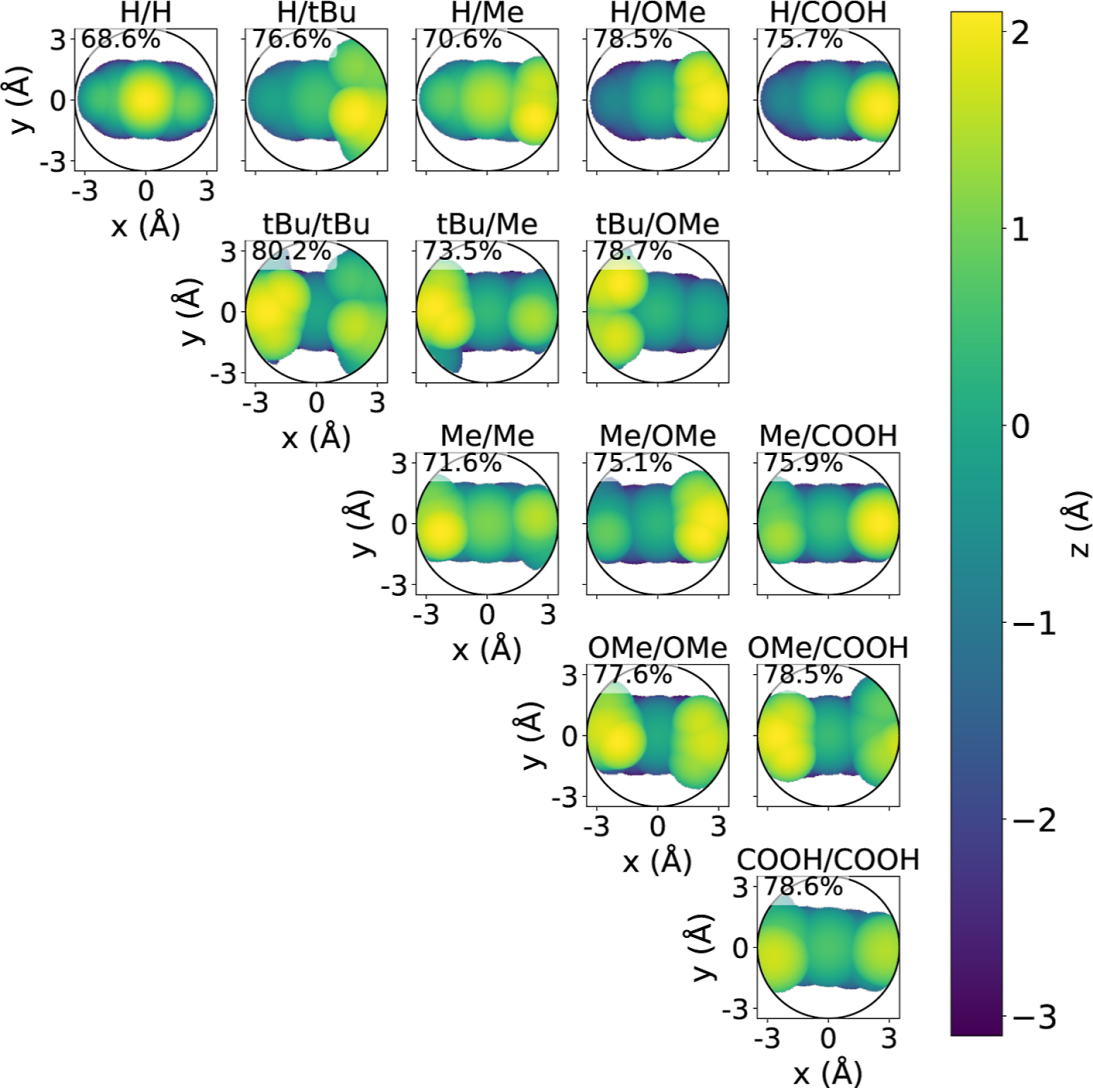
Steric maps of representative antioxidant substituent combinations. Each subplot shows the steric contour in the plane perpendicular to the O–C bond axis of the phenolic group, with the percentage indicating the buried volume. For example, H/H denotes an antioxidant radical with hydrogen substituents at both ortho positions, whereas *t*Bu/Me denotes an antioxidant radical with a *tert*-butyl group at one ortho position and a methyl group at the other.

### 3.4 Multiple Linear Regression

To evaluate the contributions of diffusivity, H-bonding, and steric hindrance to the performance metric, we performed a multivariate linear regression using normalized features (diffusion coefficient, H-bond probability, and buried volume) as predictors of the rate constant. Each feature was standardized using *z*-score normalization, where the mean of each variable is subtracted from its values and divided by its standard deviation. This process centers the data around zero with a unit variance, ensuring comparability across different scales and enabling direct interpretation of the regression coefficients.^51^ The fitted model is

1 $$\mathrm{k̂}=\beta_{0}+\beta_{1}\cdot x_{D}+\beta_{2}\cdot x_{HB}+\beta_{3}\cdot x_{BV}$$

where *k̂* is the predicted rate constant, *x*_D_ is the normalized diffusion coefficient, *x*_HB_ is the normalized H-bond probability, and *x*_BV_ is the normalized buried volume. The fitted coefficients were β_0_ = 7.54, β_1_ = 1.40, β_2_ = −2.00, and β_3_ = −1.09. The coefficient of determination for the model was *R* ^2^ = 0.65.

Figure 10 compares the regression predictions with the simulated rate constants. While the model captures the overall trend, a clear deviation appears at high rate constants, where the predictions begin to plateau. This flattening reflects a limitation of the linear model: in the high-reactivity regime, key features such as H-bonding contributions become uniformly low (Figure 6), leaving insufficient variability for the regression model to distinguish among highly reactive radicals. Consequently, the model underestimates the high rate constants. Even with this limitation, the regression coefficients remain informative. Diffusivity shows a positive contribution to reactivity (β_1_ = 1.40), indicating that higher diffusivity increases the likelihood of reaction events. Buried volume has a negative coefficient of similar magnitude (β_3_ = −1.09), indicating that increased steric hindrance around the radical site reduces the reaction rate. For H-bonding, we can only conclude that it suppresses reactivity in the low-reaction rate region, since it has a negative coefficient (β_2_ = −2.00). In the high-reaction rate region, this effect cannot be explained by H-bonding because the number of H-bonds is uniformly low there. Together, these results show how diffusion, H-bonding, and steric hindrance jointly influence the observed rate of the reverse hydrogen transfer reaction.

**Figure 10:**
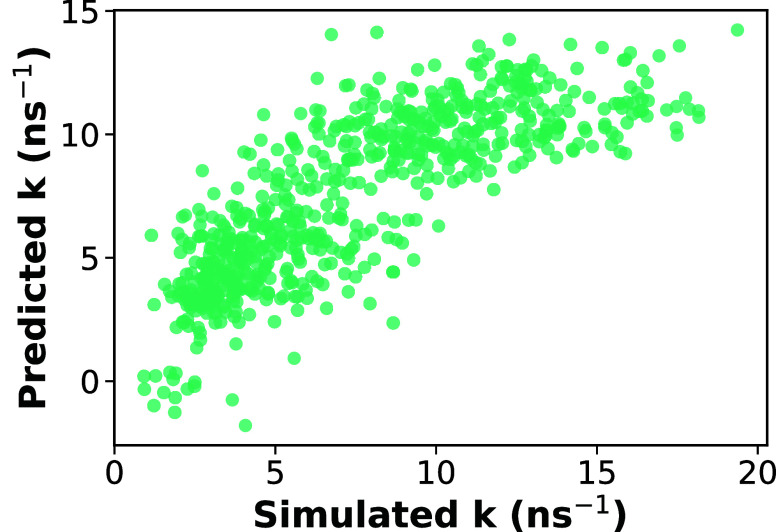
Simulated versus regression-predicted reaction rate constant obtained from the multivariate linear regression analysis. Each symbol represents an individual antioxidant radical.

To evaluate whether the regression model captures chemically meaningful trends, we examined the ranking of well-known single-ring phenolic antioxidant radicals using the predicted rate constants. The model predicts rate constants of 10.97, 12.47, and 14.25 ns^–1^ for the butylated hydroxytoluene (BHT), 2-butylated hydroxyanisole (2-BHA), and 3-butylated hydroxyanisole (3-BHA) radicals, respectively. Lower rate constants correspond to greater radical stability, which is favorable for antioxidant performance. Prior experimental studies evaluate antioxidant effectiveness through radical-scavenging behavior, where BHT is generally identified as the most effective, followed by BHA isomers.^52^ This trend is consistent with the observation that the most effective antioxidants also form highly stable antioxidant radicals.

## 4 Conclusion

This study used REACTER-based molecular dynamics to examine the stability of phenoxyl radicals formed from phenolic antioxidants in lubricants. By simulating 718 systematically generated antioxidant radicals in PAO hydroperoxides and measuring hydrogen-transfer events, we obtained an effective reaction rate constant that served as a direct indicator of antioxidant radical stability.

Analysis of the full data set focused on three molecular features, diffusivity, H-bonding, and steric hindrance around the phenoxyl oxygen atom, which together provided insight into the observed stability trends. Faster diffusion increases the chance of reactive encounters, while stronger hydrogen bonding and greater steric hindrance both reduce the likelihood of reverse hydrogen transfer. Buried volume and steric maps confirmed that bulky ortho substituents provide the most effective protection of the radical center. A simple linear regression using these descriptors showed that H-bonding, diffusion, and steric effects all contributed to the observed reactivity, with H-bonding having the strongest influence in the low reactivity region.

Overall, the results demonstrate that this framework can capture the key molecular factors that determine antioxidant-derived radical stability while remaining efficient enough to screen a large number of chemical structural variations. The trends identified here provide practical guidance for designing new phenolic antioxidants, particularly those that combine strong H-bonding capability with effective steric hindrance at the ortho positions.
